# Simulation of the Impact of Ionized Impurity Scattering on the Total Mobility in Si Nanowire Transistors

**DOI:** 10.3390/ma12010124

**Published:** 2019-01-02

**Authors:** Toufik Sadi, Cristina Medina-Bailon, Mihail Nedjalkov, Jaehyun Lee, Oves Badami, Salim Berrada, Hamilton Carrillo-Nunez, Vihar Georgiev, Siegfried Selberherr, Asen Asenov

**Affiliations:** 1 Engineered Nanosystems Group, Department of Neuroscience and Biomedical Engineering, School of Science, Aalto University, P.O. Box 12200, FI-00076 Aalto, Finland; 2 Device Modelling Group, School of Engineering, University of Glasgow, Glasgow G12 8LT, Scotland, UK; Cristina.MedinaBailon@glasgow.ac.uk (C.M.-B.); jaehyun1986.lee@gmail.com (J.L.); Oves.Badami@glasgow.ac.uk (O.B.); Salim.Berrada@glasgow.ac.uk (S.B.); Hamilton.Carrillo-Nunez@glasgow.ac.uk (H.C.-N.); Vihar.Georgiev@glasgow.ac.uk (V.G.); Asen.Asenov@glasgow.ac.uk (A.A.); 3 Institute for Microelectronics, TU Wien, Gusshausstrasse 27-29/E360, 1040 Wien, Austria; mixi@iue.tuwien.ac.at (M.N.); Selberherr@TUWien.ac.at (S.S.)

**Keywords:** nanowire field-effect transistors, silicon nanomaterials, charge transport, one-dimensional multi-subband scattering models, Kubo–Greenwood formalism, schrödinger-poisson solvers

## Abstract

Nanowire transistors (NWTs) are being considered as possible candidates for replacing FinFETs, especially for CMOS scaling beyond the 5-nm node, due to their better electrostatic integrity. Hence, there is an urgent need to develop reliable simulation methods to provide deeper insight into NWTs’ physics and operation, and unlock the devices’ technological potential. One simulation approach that delivers reliable mobility values at low-field near-equilibrium conditions is the combination of the quantum confinement effects with the semi-classical Boltzmann transport equation, solved within the relaxation time approximation adopting the Kubo–Greenwood (KG) formalism, as implemented in this work. We consider the most relevant scattering mechanisms governing intraband and multi-subband transitions in NWTs, including phonon, surface roughness and ionized impurity scattering, whose rates have been calculated directly from the Fermi’s Golden rule. In this paper, we couple multi-slice Poisson–Schrödinger solutions to the KG method to analyze the impact of various scattering mechanisms on the mobility of small diameter nanowire transistors. As demonstrated here, phonon and surface roughness scattering are strong mobility-limiting mechanisms in NWTs. However, scattering from ionized impurities has proved to be another important mobility-limiting mechanism, being mandatory for inclusion when simulating realistic and doped nanostructures, due to the short range Coulomb interaction with the carriers. We also illustrate the impact of the nanowire geometry, highlighting the advantage of using circular over square cross section shapes.

## 1. Introduction

As the conventional CMOS technology is approaching the scaling limits, different technologies and materials are being studied extensively to extend the end of the Roadmap. One possible solution is based on different injection mechanisms such as the tunnel field effect transistor (TFET), whose most promising characteristic is reaching sub-60 mV/dec subthreshold swings (SS) [1,2,3]. On the other hand, the utilization of multiple gates surrounding the channel (for instance the double-gate FinFET devices in comparison to the planar fully Depleted Silicon-On-Insulator (FDSOI) devices [4,5]) improves charge control in the channel (minimizing the short-channel effects), improves the transport properties and includes the possibility of using material and strain engineering to improve the device performance. In this context, the interest in gate-all-around (GAA) nanowire transistors (NWTs) is soaring, since they are considered as a potential replacement of the FinFET CMOS technology to the ultimate scaling limits (down to 5 nm). The benefits of NWT technology are discussed extensively in literature [6,7,8,9]. In the simulation and optimisation of NWTs, the methods incorporating quantum confinement effects into semi-classical transport approaches [10,11,12] are becoming very attractive thanks to their lower computational demand, as compared to the purely quantum transport and atomistic models [13,14,15]. The continuous interest in these models is prompted by the need to accurately model silicon (Si) based NWTs, whose electronic transport properties are conditioned by multi-subband scattering at diameters smaller than 7–8 nm [12].

The importance of spatial confinement in advanced CMOS devices whose downscaling follows the Moore’s law, known as More Moore transistors (e.g., NWTs or FinFETs), is significant. In such transistors, the basic properties of electronic charge transport, derived under the assumption of an ideal periodic crystal, must be revised. In this case, the Bloch theorem is no longer applicable, as charge carriers are confined typically in a cross section perpendicular to the transport direction. In such circumstances, the charge carriers (electrons or holes) cannot be considered point-like particles and their motion is constrained in the confinement plane. Also, as a result of the position-momentum uncertainty relations, the momentum of a localized carrier is not well defined. Instead, the energy is quantized into subbands and the momentum conservation is only valid in the transport direction. The rates for the corresponding multi-subband scattering processes are altered by the overlap factor of the eigenfuctions of the subbands involved in the carrier transition events [12].

Significant efforts have been invested in calculating the mobility of silicon nanowires, employing mainly the Kubo–Greenwood (KG) formalism [10,11]. Other methods have also been used to a lesser extent, such as multi-subband Monte Carlo simulators, one-dimensional (1D) multi-subband Boltzmann transport equation (BTE) solvers [12,16], and atomistic simulations [13]. Despite this progress, more work is warranted to evaluate the performance potential of NWTs with silicon channels at the scaling limit. In this work, we employ a newly developed simulation framework using the Poisson-Schrödinger solver of the Technology Computer Aided Design (TCAD) simulator GARAND [17], coupling a three-dimensional (3D) Poisson solver to two-dimensional (2D) Schrödinger solutions in the discretization planes normal to the transport direction. GARAND is in turn coupled to a standalone KG module performing multi-subband calculations of the 1D scattering rates and calculating the mobility, in order to explore the impact of the overlap factor, the different scattering mechanisms—with special attention given to ionized impurity (II) scattering—and the impact of the cross sectional shape on the electron mobility in silicon NWTs. This work focuses studying the electron transport properties in *n*-type devices (see e.g., [10,11,12,13,16]), as the purpose of this study is to illustrate the impact of scattering mechanisms in nanowire transistors, in general. However, our model can be readily extended to study *p*-type devices, and further research is warranted to explore hole transport effects [14,18] in these 1D structures. The innovation in this work is two-fold: the unique combination of simulation tools and models integrated together to accurately model confined transport properties in very small diameter NWTs, and the extensive study of physical effects impacting transport in these nanotechnology devices. As compared to other established simulators [12,13,16], our method incorporates quantum confinement effects and provides reliable low field nanowire mobility, in a transparent manner, while requiring relatively low computational cost and simulation time.

The structure of this paper is organized as follows. Section 2 gives a general overview of the mobility simulation approach, describing the simulation of the device electrostatic and multi-subband properties, and providing the details of the considered 1D (phonon, surface roughness, and ionized impurity) scattering mechanisms as well as their rate formulas. Moreover, the different equations needed in the KG formalism and the Matthiessen rule are expressed. Section 3 outlines the main results and their discussion, including a meticulous analysis of the impact of the ionized impurity scattering mechanism on the total electron mobility.

## 2. Simulation Method and Physics

### 2.1. Simulation Framework

**Figure 1:**
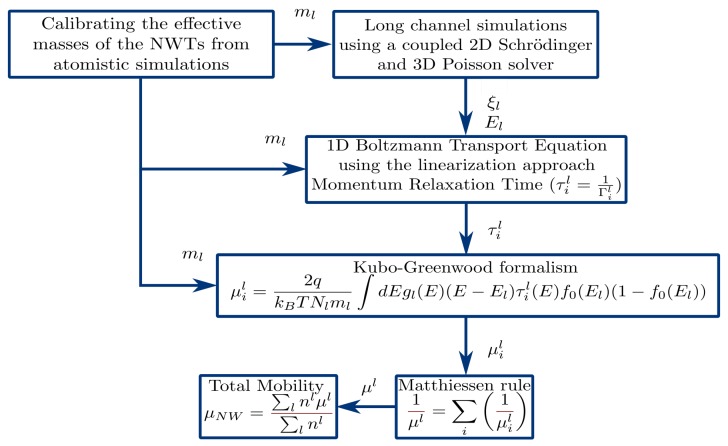
A flowchart illustrating the simulation framework and the corresponding steps needed to calculate the total mobility. *l* is the subband index; $m_{l}$, $\xi_{l}$, and $E_{l}$ are the calibrated effective masses, the wavefunction, and the energy level for the *l*th subband, respectively; *i* is the *i*-th scattering mechanism; $\tau_{i}^{l}$, $\Gamma_{i}^{l}$, and $\mu_{i}^{l}$ are the relaxation time, scattering rate, and mobility, respectively, for the *i*th mechanism and *l*th subband; $\mu_{NW}$ is the total mobility for a particular NW structure.

The simulator used in this work is based on the long-channel, low-bias mobility model, whose capabilities have already been established when analyzing the impact of scattering mechanisms on nanowire performance [19]. The simulation process proceeds in three steps. In the first step, we use the coupled 3D Poisson–2D Schrödinger solver in GARAND to evaluate the electric potential and field distributions at the cross section area of a gated (long channel) NWT, and to calculate the electron densities and the details of the electronic subbands (eigenfunctions and eigenvalues) in the NWT cross section normal to the transport direction. In the second step, we utilize the potential distribution, and the corresponding eigenfunctions (and relevant subband details) to calculate the 1D transition rates for the dominant scattering mechanisms in silicon, including the modified acoustic phonon, optical phonon, ionized impurity [18] and surface roughness scattering [20]. In the final step, we use the set of multi-subband scattering mechanism rates to calculate the scattering-limited mobilities of interesting NWT structures, by adopting the KG formalism solving for the (semi-classical) BTE within the relaxation time approximation [10,11]. Before initiating the simulation process, the confinement and transport masses are extracted from first principle simulations, accounting for the impact of the cross-section and diameters of the nanowire. The effective masses are extracted from sp${}^{3}$d${}^{5}$s${}^{*}$ tight-binding simulations with a Boykin parameter set [21]. Taking into account these transport effective masses, multiple cross sections of the device are simulated in GARAND. The flowchart in Figure 1 illustrates the simulation procedure. The simulation framework provides reliable mobilities at low-field near-equilibrium conditions in transistors exhibiting strong confinement effects, such as the NWTs considered here. While the main purpose of this work is to study silicon nanowires, the simulator can be easily improved to simulate nanowires based on related alloys, such as SiGe, once a model for disordered alloy scattering is implemented.

### 2.2. Subband Details and Electrostatics

**Figure 2:**
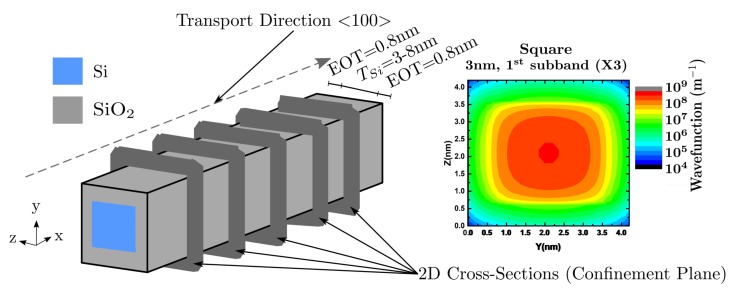
Coupling the 2D solution of the Schrödinger equation in multiple slices to a 3D Poisson solution in a structure based on a Si gate-all-around nanowire channel FET.

The simulator self-consistently couples a 1D BTE model with the solution of the Poisson and Schrödinger equations in a cross section of the NWT normal to the transport direction. It involves intensive computations to determine the multi-subband energies and the eigenfunctions, and the resulting potential distribution in the NWT. This implies that the numerical aspects are an important factor for the feasibility of the simulation model, which delivers a compromise between physical accuracy and computational efficiency. GARAND couples the 2D solution of the Schrödinger equation in multiple slices (i.e., the cross section areas of the long channel) to a 3D Poisson solution in the simulated Si gate-all-around NWT, as shown in Figure 2. The solution provides the parameters and fields needed for the scattering rates and KG mobility calculations, including (i) the electric potential and field distributions used e.g., for surface roughness scattering, (ii) the details of the electronic subbands (eigenfunctions and eigenvalues) and (iii) subband electron densities.

### 2.3. 1D Scattering Rates

The scattering rates for the electron interaction mechanisms with (acoustic and optical) phonons, impurities and surface roughness used in this study, which are considered as silicon mobility limiting mechanisms, are presented in this section. The derivation of the rates is performed within the ellipsoidal non-parabolic valleys bandstructure approximation. The rates for the scattering mechanisms are derived from the Fermi’s Golden Rule, using the time-dependent perturbation theory and assuming that the transitions between two states occur instantaneously.

#### 2.3.1. Acoustic Phonon Scattering

We consider acoustic phonon scattering mechanisms in the elastic parabolic equipartition approximation, within the short wave vector limit [22]. After extensive derivations, the scattering rate is given by:

(1) $$\begin{array}{c} \Gamma \left(ac,l,k\right)=\frac{|D_{ac}{|}^{2}k_{B}T}{\rho \hbar {\bar{u}}^{2}}\frac{m}{\hbar^{2}}\sum_{l^{\prime }}\left[\int ds|\xi_{l}{\left(s\right)|}^{2}{\left|\xi_{l^{\prime }}\left(s\right)\right|}^{2}\right]\times \theta \left(\epsilon \left(k\right)+\Delta E_{l^{\prime }}\right)\left(\frac{1}{|q_{1}+k|}+\frac{1}{|q_{2}+k|}\right), \end{array}$$

with

(2) $$q_{1/2}=-k\pm \sqrt{k^{2}+\frac{(E_{l}-E_{l^{\prime }})2m}{\hbar^{2}}}.$$

Here, $D_{ac}$ is the deformation potential, $k_{B}$ is the Boltzmann constant, *T* is the lattice temperature, ρ is the material density, *ℏ* is the reduced Planck’s constant, $\bar{u}$ is the speed of sound, *m* is the electron effective mass. Also, *l* and $l^{\prime }$ refer to the initial and final electron subbands, s are vectors normal to the transport direction, ξ are the wavefunctions at the given subband, θ represents the heaviside step function, ϵ(k) is the kinetic energy for a wavevctor magnitude *k*, and $\Delta E_{l^{\prime }}=E_{l^{\prime }}-E_{l}$ is the energy separation between subbands *l* and $l^{\prime }$.

#### 2.3.2. Optical Phonon Scattering

The energies of the different branches of the deformation potential optical phonons are approximated with constants, as used in most of the standard approaches. Accordingly, the scattering rate can be written as:

(3) $$\Gamma \left(op,j,l,k\right)=\frac{|D_{op,j}{|}^{2}}{2\rho \omega_{j}}\sum_{l^{\prime }}\left[\int ds|\xi_{l}{\left(s\right)|}^{2}{\left|\xi_{l^{\prime }}\left(s\right)\right|}^{2}\right]\int dqG\left(q\right),$$

where

$$\begin{array}{c} \int dqG\left(q\right)=\frac{n_{j}\theta \left(\epsilon \left(k\right)+\Delta E_{l^{\prime }j}^{+}\right)m}{\hbar^{2}}\left(\frac{1}{|q_{1}+k|}+\frac{1}{|q_{2}+k|}\right)\\ +\frac{\left(n_{j}+1\right)\theta \left(\epsilon \left(k\right)+\Delta E_{l^{\prime }j}^{-}\right)m}{\hbar^{2}}\left(\frac{1}{|q_{3}+k|}+\frac{1}{|q_{4}+k|}\right) \end{array}$$

with:

(4) $$q_{1/2}=-k\pm \sqrt{k^{2}+\frac{(E_{l}+\hbar \omega_{j}-E_{l^{\prime }})2m}{\hbar^{2}}},q_{3/4}=-k\pm \sqrt{k^{2}+\frac{(E_{l}-\hbar \omega_{j}-E_{l^{\prime }})2m}{\hbar^{2}}},$$

and

(5) $$\Delta E_{l^{\prime }j}^{+}=E_{l}-E_{l^{\prime }}+\hbar \omega_{j}\;\mathrm{and}\;\Delta E_{l^{\prime }j}^{-}=E_{l}-E_{l^{\prime }}-\hbar \omega_{j}.$$

Here, $n_{j}$ is the equilibrium phonon number, *j* refers to the phonon mode and $\omega_{j}$ is the phonon energy.

#### 2.3.3. Surface Roughness Scattering

Surface roughness scattering is most pronounced when confinement keeps electrons close to non-ideal interfaces. The extent of the interaction with surface imperfections is dependent on the force normal to the interface, and the statistical description of the interface roughness [20]. Assuming *x* is the direction of transport along the nanowire, the perturbation Hamiltonian can be written as:

(6) $$H^{\prime }=eE_{y}\left(s,x\right)\Delta_{y}\left(x\right)+eE_{z}\left(s,x\right)\Delta_{z}\left(x\right),$$

where Δ(y) and Δ(z) are the corresponding deviations of the silicon nanowire surface from the ideal surface, and $E_{y}$ and $E_{z}$ are the electric field components in the cross section normal to the direction of transport. The corresponding scattering rate can be derived as:

(7) $$\Gamma \left(sr,l,k\right)=\sum_{l^{\prime }}\frac{e^{2}}{\hbar }{\left|N_{E}\left(l,l^{\prime }\right)\right|}^{2}D^{2}\frac{m}{\hbar^{2}}\left(\frac{2\sqrt{2}\lambda }{\left(2+\lambda^{2}{\left(k-k_{1}^{\prime }\right)}^{2}\right)}\frac{1}{|k_{1}^{\prime }|}+\frac{2\sqrt{2}\lambda }{\left(2+\lambda^{2}{\left(k-k_{2}^{\prime }\right)}^{2}\right)}\frac{1}{|k_{2}^{\prime }|}\right)\theta \left(\epsilon_{f}\right),$$

where

(8) $$N_{E}\left(l,l^{\prime }\right)=N_{E_{x,y}}\left(l,l^{\prime }\right)=\int ds\xi_{l^{\prime }}^{*}\left(s\right)E_{x,y}\left(s\right)\xi_{l}\left(s\right).$$

Here, the indices *x* and *y* indicate that the integral in Equation (8) is evaluated separately for the two components of the field. The θ function comes from the requirement for existence of the square root $k_{1,2}^{\prime }=\pm \sqrt{\frac{2m}{\hbar^{2}}\left(E_{l}+\epsilon \left(k\right)-E_{l^{\prime }}\right)}=\pm \sqrt{\frac{2m}{\hbar^{2}}\epsilon_{f}}$. The result is generalized for the particular *y* and *z* components of Equation (6). Also, *D* is the root mean square of the variance of Δ, and λ is the correlation length. In this work, *D* and λ are set to 0.48 nm and 1.3 nm, respectively.

#### 2.3.4. Ionized Impurity Scattering

The screened Coulomb potential of an impurity at position (S,0) for an electron at (s,z) is given by [18]:

(9) $$V\left(r\right)=\frac{Z_{I}e^{2}}{4\pi \epsilon \sqrt{{\left(S-s\right)}^{2}+z^{2}}}e^{-\left(\sqrt{{\left(S-s\right)}^{2}+z^{2}}\right)/L_{D}},$$

where, for a multi-subband case, the screening (Debye) length $L_{D}$ is given by:

(10) $$L_{D}^{2}=\frac{kT\epsilon }{e^{2}n_{0}}\frac{\sum_{l}F_{-1/2}\left(\left(E_{l}-E_{F}\right)/kT\right)}{\sum_{l}F_{-3/2}\left(\left(E_{l}-E_{F}\right)/kT\right)}.$$

Here $F_{n}$ is the Fermi integral of order *n*, and $n_{0}$ is the equilibrium electron density. The scattering rate is given by

(11) $$\begin{array}{c} \Gamma \left(imp,S^{\prime },Z^{\prime };l,k\right)=\frac{2m}{\hbar^{3}}N_{I}{\left(\frac{Z_{I}e^{2}}{4\pi \epsilon }\right)}^{2}\frac{1}{{\left(2\pi \right)}^{3}}\sum_{j=1}^{2}\frac{1}{2|q_{j}+k|}\sum_{l^{\prime }}{\left|\int dq_{S}\int \frac{4\pi d\mathrm{sv}\xi_{l^{\prime }}^{*}\left(s\right)\xi_{l}\left(s\right)e^{iq_{S}s}}{q_{S}^{2}+q_{j}^{2}+{\left(1/L_{D}\right)}^{2}}\right|}^{2}. \end{array}$$

In this case:

(12) $$q_{1/2}=-k\pm \sqrt{k^{2}-\frac{2m(E_{l^{\prime }}-E_{l})}{\hbar^{2}}}.$$

It is noteworthy that, for both surface roughness and ionized impurity scattering, we assume elastic and intra-valley transitions.

### 2.4. Mobility Calculation

The mobility, calculated from the set of multi-subband scattering mechanisms discussed above, is based on the KG theory applied to a confined 1D electron gas. The semi-classical simulation of the transport properties of a 1D electron gas requires the solution of the BTE, which is not straightforward in complex structures. The KG formalism employed in this study is based on the relaxation times approximation, which represents a solution of the linearized BTE. The KG formalism provides accurate mobility values at low-field near-equilibrium conditions, based on the rates of the relevant scattering mechanisms governing multi-subband transitions in quantum wires. The developed mobility theory involves a set of approximations used to define the momentum relaxation time of the 1D electron gas. For a scattering mechanism *i* and subband *l*, the mobility is calculated by

(13) $$\mu_{i}^{l}=\frac{2q}{k_{B}TN_{l}m_{l}}\int dEg_{l}\left(E\right)\left(E-E_{l}\right)\tau_{i}^{l}\left(E\right)f_{0}\left(E\right)\left(1-f_{0}\left(E\right)\right)$$

where $m_{l}$ is the subband effective mass, $\tau_{i}^{l}$ is the relaxation time, $g_{l}$ is 1D density of states, $f_{0}$ is the Fermi-Dirac function, and $N_{l}$ is the ‘1D’ electron concentration of the *l*th subband given by

(14) $$N_{l}=N_{C1D}\left[-F\{\frac{1}{2},-\exp\left(\frac{E_{F}-E_{l}}{k_{B}T}\right)\}\right].$$

In this case, $N_{C1D}$ is the effective 1D density of states, F is the Fermi integral of order 1/2, and $E_{F}$ is the Fermi level. Since several scattering phenomena are considered at the same time in this study, the total mobility $\mu_{l}$ for each subband *l* must include the cumulative effect of all scattering probabilities by applying the so-called Matthiessen’s rule:

(15) $$\frac{1}{\mu^{l}}=\sum_{i}\left(\frac{1}{\mu_{i}^{l}}\right).$$

The average mobility of a particular nanowire structure must be calculated accounting for all the subbands:

(16) $$\mu_{NW}=\frac{\sum_{l}n^{l}\mu^{l}}{\sum_{l}n^{l}},$$

where $n^{l}$ is the electron density in the given subband.

## 3. Results and Discussion

The simulated GAA structure is illustrated in Figure 2. It consists of a square (default structure) or a circular Si nanowire of a [100] channel orientation, surrounded by a thin silicon dioxide layer, with an equivalent oxide thickness (EOT) of 0.8 nm, and a gate contact. The nanowire diameter is varied from 8 nm, where the NWT has a bulk-like behaviour, down to 3 nm, where the device’s behaviour is fully quantum. This work additionally presents a deeper analysis of the characteristics of a 3 nm diameter NWT, as this is the smallest possible thickness before mobility sharply falls due to scattering from confinement fluctuation.

As is known, FinFETs and NWTs have the advantage of tolerating very low doping, resulting in low statistical variability [23]. However, many NWT designs rely e.g., on the formation of junctions between the heavily doped source and drain contacts and the undoped 1D channel, imposing severe constraints on doping techniques to fulfill the need for ultrasharp source and drain junctions. In this context, understanding the impact of charged impurity scattering on the NWT behaviour is of great interest. The presented simulations are carried out for a wide range of equivalent ionized impurity/doping concentrations $N_{I}$, from very low values ($10^{16}$ cm${}^{-3}$) to values as high as $10^{20}$ cm${}^{-3}$. The practical purpose of such analysis is to illustrate how e.g., the unintentional presence of discrete charged impurities [24] or intentional doping during the fabrication process of Si NW channels [25,26] (for example for threshold voltage tuning), can affect the device performance. Indeed, scattering by ionized impurity (Coulombic) centers is suggested to be very important in very thin Si NWs with diameters below 5 nm, due to their small cross section [18,27].

**Figure 3:**
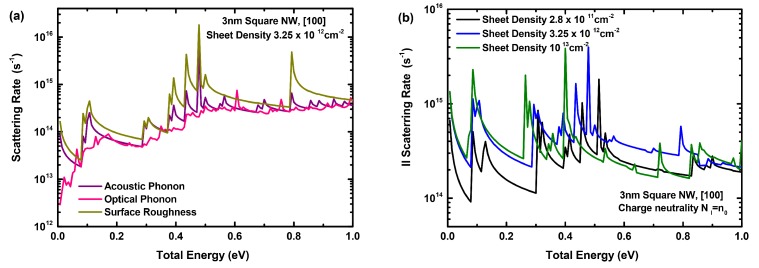
(**a**) The rates for acoustic and optical phonons, as well as surface roughness scattering as a function of the total energy, for a sheet density of $3.25\times 10^{12}\;$cm${}^{-2}$. (**b**) The rates for ionized impurity scattering, as a function of the total energy and different sheet densities, assuming charge neutrality. The results are for a 3 nm diameter square nanowire structure.

Figure 3a shows the variation of the rates for acoustic and optical phonons, as well as surface roughness scattering as a function of the total energy, for the above NWT with a diameter of 3 nm and at a moderately high sheet density of $3.25\times 10^{12}\;$cm${}^{-2}$. In this paper, we assume that the sheet density $N_{s}$ in the nanowire (of any shape) is related to the volumetric density *N* via $N=N_{s}/L_{p}$, where $L_{p}$ is the nanowire perimeter. Figure 3b shows the corresponding variation of the ionized impurity rates at channel doping concentrations varying from ∼2 $\times \;10^{17}\;$cm${}^{-3}$ to $∼10^{19}\;$cm${}^{-3}$, and the corresponding free carrier sheet densities assuming charge neutrality. The carrier sheet density is controlled directly by varying the bias of the gate contact $V_{G}$, increasing as $V_{G}$ is raised. The rates are shown for all the subbands in the three two-fold conduction valleys of interest in the [100] oriented NWT channel. While the impact of surface roughness scattering at low carrier sheet densities is small, its rate becomes significant at the charge density of $3.25\times 10^{12}\;$cm${}^{-2}$ as highlighted in Figure 3a. While the acoustic phonon scattering rate is in general higher than the optical phonon scattering rate, the impact of the former mechanism on mobility is expected to be much more pronounced, as its rate is many times higher at the lowest subband where most electrons are located. As for ionized impurity, its rate increases at higher impurity concentrations, as expected. For the impurity concentrations mentioned above (>∼10${}^{17}\;$cm${}^{-3}$), the II rate is generally highest compared to all other scattering mechanisms at the lowest subband where most electrons are located, making II scattering one of the dominant factors impacting negatively NWT mobility, as discussed in more detail below.

**Figure 4:**
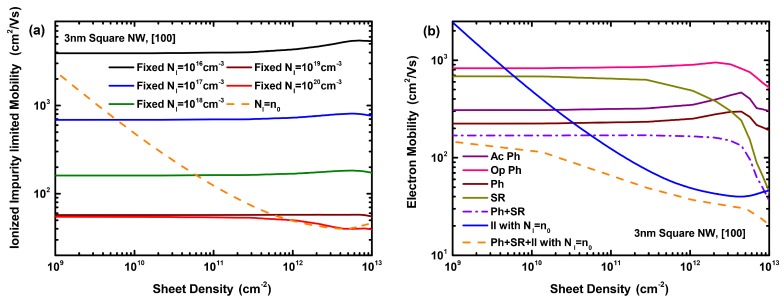
(**a**) Ionized impurity limited electron mobility as a function of the sheet density considering different fixed ionized impurity densities as well as charge neutrality. (**b**) Electron mobility as a function of the sheet density considering acoustic, optical, total phonon, surface roughness, and ionized impurity (assuming charge neutrality) scattering separately, as well as the combined mechanisms. The results are for a 3 nm diameter square nanowire structure.

Figure 4a shows the ionized impurity limited electron mobility as a function of the sheet density considering various ionized impurity densities ($N_{I}$) as well as charge neutrality ($N_{I}$ = $n_{0}$). As the II scattering rate is directly proportional to the impurity density, the II limited mobility decreases according to 1/$N_{I}$. This is reflected in the curve showing the rate variation for the charge neutrality case where the mobility curve has a hyperbola-like shape when $N_{I}$ is varied. The II-limited mobility falls from 4000 cm${}^{2}$/Vs at $N_{I}$ = $10^{16}$ cm${}^{-3}$ to below 60 cm${}^{2}$/Vs at $N_{I}$ = $10^{20}$ cm${}^{-3}$, indicating that this scattering mechanism is dominant at high $N_{I}$, adversely affecting the mobility of the nanowire structure. Figure 4b shows the electron mobility as a function of the sheet density considering acoustic (Ac Ph), optical (Op Ph), total phonon (Ph), surface roughness (SR), and ionized impurity (assuming charge neutrality) scattering separately, as well as the combined mechanisms. The phonon limited mobility is almost constant up to a sheet density of ∼3 $\times \;10^{12}$ cm${}^{-2}$, and starts to vary and visibly decrease at higher densities. The surface roughness limited mobility is relatively constant up to a sheet density of ∼3 $\times \;10^{11}$ cm${}^{-2}$. However, it starts to decrease dramatically as the sheet density is increased reaching a value much lower than the phonon-limited mobility at ∼10${}^{13}$ cm${}^{-2}$; this significant decrease is partly associated with the higher magnitudes of the field components perpendicular to the transport direction. As expected, II scattering may degrade the total mobility especially at sheet densities beyond ∼10${}^{11}$ cm${}^{-2}$. At the highest sheet density considered, the phonon-limited mobility is 200 cm${}^{2}$/Vs; with surface roughness and II scattering included, the mobility falls down to 50 cm${}^{2}$/Vs and 20 cm${}^{2}$/Vs, respectively. It is noteworthy that, at very high sheet densities (>$4\times 10^{12}$ cm${}^{-2}$) and impurity concentrations (>$10^{20}$ cm${}^{-3}$), the II scattering starts to decrease again, due to screening, explaining the visible increase in the II-limited mobility shown at these densities in Figure 4.

**Figure 5:**
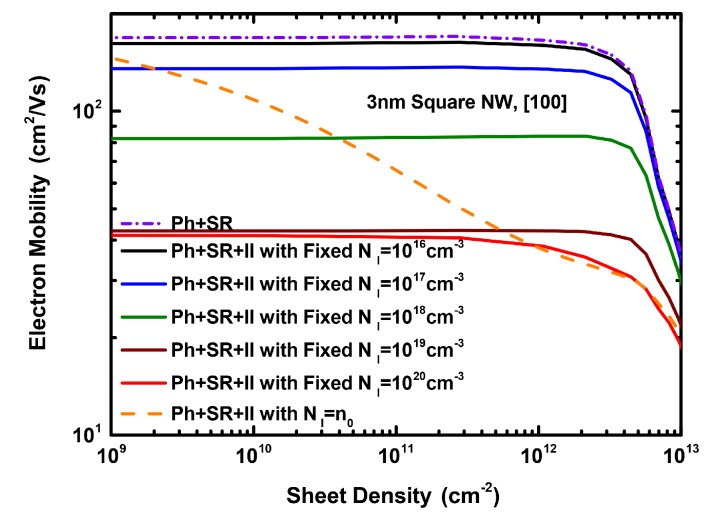
The electron mobility as a function of the sheet density considering the combined total phonon and surface roughness (Ph + SR) scattering rates, as well as the total scattering (Ph + SR + II). The results are for different fixed ionized impurity densities and the charge neutrality case, for a 3 nm diameter square NW with a [100] channel orientation.

**Figure 6:**
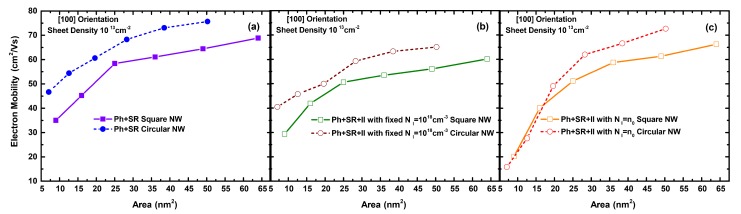
The electron mobility as a function of the NW cross sectional area, considering the impact of the combined total phonon and surface roughness (Ph + SR) scattering effects (**a**), as well as all present scattering mechanisms (Ph + SR + II) with a (**b**) fixed ionized impurity density ($N_{I}=10^{18}$ cm${}^{-3}$) and (**c**) the charge neutrality case. The results are for both square and circular NWs ([100] orientation) and a sheet density of $10^{13}\;$ cm${}^{-2}$.

Figure 5 shows how the total electron mobility varies, as a function of sheet density, for different ionized impurity densities and the charge neutrality case. Figure 5 confirms the role of II scattering in degrading the total NWT channel mobility for a large range of sheet densities. Figure 6 illustrates the variation of the nanowire mobility with the nanowire cross sectional area, for three cases: when including (a) only phonon and SR (Ph + SR) scattering, and phonon and SR scattering alongside ionized impurity scattering with (b) a fixed (moderately high) impurity concentration ($N_{I}=10^{18}$ cm${}^{-3}$) and (c) the charge neutrality case. In all cases, circular nanowires exhibit generally higher mobilities than the square nanowires for the same area. Albeit, such an advantage is less significant when including II scattering, especially at higher impurity concentrations as is the case for lower diameters (e.g., 3 nm and 4 nm) in the charge neutrality case.

## 4. Conclusions

We have developed and employed a unique multi-subband simulator, coupling the KG formalism to an efficient Poisson–Schrödinger solver, to provide a deeper insight into charge transport in NWTs. We have explored the effect of various scattering mechanisms and nanowire shapes on the electron mobility of gate-all-around Si NWTs. This paper emphasizes the importance of accounting correctly for quantum confinement and multi-subband transistions, to provide a reliable prediction of device performance, and the role of phonon, surface roughness and ionized impurity scattering in degrading the nanowire mobility. Results show that acoustic phonon scattering is the dominant phonon scattering mechanism, and that the influence of surface roughness scattering becomes significant compared to phonon-mediated mechanisms only at very high mobile charge densities. Also, we carried out a thorough analysis of the (negative) impact of the ionized impurity scattering, showing that this mechanism dominates in realistic and doped structures, at impurity concentrations above ∼10${}^{18}\;$cm${}^{-3}$. Further analysis shows that circular nanowires give higher mobilities compared to square nanowires, at a given cross-sectional area, but such benefit is minimized at high impurity concentrations when including II scattering. The presented simulation framework is a valuable tool for technology option screening for ultra-scaled NWTs, in terms of performance.
